# Attractiveness of Green Stink Bugs *Nezara* spp. to Ultraviolet-Based Multichromatic Light Traps: Synergistic Effects of Ultraviolet and Blue Light

**DOI:** 10.3390/insects17030270

**Published:** 2026-03-03

**Authors:** Nobuyuki Endo, Mantaro Hironaka, Yoshiyuki Honda, Hiroaki Takeuchi, Kazuki Shibuya

**Affiliations:** 1 Institute for Plant Protection, National Agriculture and Food Research Organization (NARO), Tsukuba 305-8666, Japan; shibuya.kazuki336@naro.go.jp; 2 Department of Bioproduction Science, Ishikawa Prefectural University, Nonoichi 921-8836, Japan; 3 Yamaguchi Prefectural Agriculture & Forestry General Technology Center, Yamaguchi 753-0231, Japan; honda.yoshiyuki.01@pref.yamaguchi.lg.jp; 4 Central Region Agricultural Research Center, National Agriculture and Food Research Organization (NARO), Joetsu 943-0193, Japan; takeuchi.hiroaki572@naro.go.jp

**Keywords:** phototaxis, wavelength composition, light trap, *Nezara viridula*, *Nezara antennata*

## Abstract

Numerous insect species exhibiting positive phototaxis are strongly attracted to ultraviolet (UV) light. However, several heteropteran stink bugs, including *Nezara viridula* (L.) and its congener *Nezara antennata* Scott, show stronger attraction to traps combining UV and green light than to monochromatic UV light traps. To examine the role of visible light wavelengths in enhancing UV attraction, we evaluated the attractiveness of blue (469 nm), green (523 nm), orange (613 nm), and red (632 nm) light in combination with UV light (396–400 nm), as well as a monochromatic UV light source, under field conditions targeting *Nezara* bugs. Traps combining UV and blue light captured nearly three times more *Nezara* bugs than UV-only light traps. Conversely, traps combining orange or red and UV light captured equal to or fewer bugs than monochromatic UV light traps, indicating no enhancement in attraction with these color combinations. Furthermore, monochromatic blue light alone showed very weak attractiveness, indicating that blue light synergistically enhanced the attractiveness of UV light to bugs. Strong attractiveness to traps combining UV and green light was confirmed in the lepidopteran moth *Pleuroptya ruralis* (Scopoli), suggesting that multiwavelength light sources may be effective in attracting insect species beyond Heteroptera. These findings highlight the value of multiwavelength light traps, particularly traps combining UV and blue light, for improving stink bug monitoring and pest management.

## Simple Summary

Many light-attracting insects are attracted to ultraviolet (UV) light. However, certain stink bugs, including the *Nezara* species, show a stronger preference for light sources combining UV with green light than for UV light alone. To investigate this, we tested whether adding visible light wavelengths to UV light enhanced capture efficiency. We compared the number of *Nezara* bugs captured in traps combining four visible light sources (blue, green, orange, and red) with UV light against traps using only UV light. Traps combining UV and blue light captured approximately three times more stink bugs than UV-only traps. Interestingly, blue light alone attracted very few bugs, indicating that it enhances UV light attraction rather than acting independently. In contrast, combining UV with orange or red light did not increase capture rates relative to UV-only traps, indicating that these wavelengths do not enhance the attractiveness of UV light. These findings strongly indicate that, for certain insect species, combining UV with specific visible wavelengths, such as blue, can be substantially more effective than UV light alone. This multiwavelength approach may improve trap effectiveness and inform the development of optimized pest control strategies.

## 1. Introduction

The southern green stink bug *Nezara viridula* (L.) (Hemiptera: Pentatomidae) is a cosmopolitan pest found in tropical and temperate locations across the Americas, Africa, Asia, Australia, and Europe, where it imposes considerable economic impacts on numerous crops worldwide [1,2]. In Japan, it primarily damages soybean and rice crops [3,4]. In the past two decades, its range has expanded northward in Japan, possibly because of global warming [5,6,7]. At the northern limit of its distribution range, *N. viridula* coexists with a congeneric species, the oriental green stink bug *Nezara antennata* Scott [5,6,7], which is prevalent across Japan [8]. In these sympatric distribution areas, *N. viridula* often replaces *N. antennata* [3,6,9]. This phenomenon has attracted considerable ecological interest.

Throughout Japan, automatic daily monitoring light traps are used to monitor the occurrence of pest insects, such as stink bugs and plant hoppers [10,11,12,13]. *Nezara* bugs exhibit positive phototaxis, making light traps effective for monitoring their population dynamics [14]. Incandescent or mercury lamps are commonly used as light sources in these traps. However, many countries have instituted prohibitions on the production of mercury lamps, reflecting a global trend toward their elimination [15]. In addition, many manufacturers in Japan have already discontinued the production of incandescent lamps because of their poor energy efficiency. The anticipated use of light-emitting diodes (LEDs) as an alternative light source is due to their energy efficiency and extended lifespan [10].

The attractiveness of light to insects is directly affected by the wavelength composition of the light source. Many nocturnal insects are attracted to light sources that emit high levels of ultraviolet (UV) light [16,17]. *Nezara* bugs are also more attracted to UV-rich mercury lamps than to incandescent ones [18]. Furthermore, in laboratory preference experiments, *N. viridula* is more strongly attracted to UV light than to visible light, with no significant differences observed between adult males and females in their responsiveness to these wavelengths [19]. These results indicate that UV light effectively attracts *Nezara* bugs. Meanwhile, our previous research revealed that combining green light, which is hardly attractive on its own, with UV light synergistically enhances insect attraction [20].

Many insects, such as those in the order Hemiptera, possess compounds with three types of photoreceptor cells that respond to UV, blue, and green wavelengths [21,22]. For adult *N. viridula*, physiological studies using electroretinography (ERG) have shown retinal responses to wavelengths ranging from 320 to 620 nm, suggesting the presence of three photoreceptor types [19]. In contrast, behavioral and modeling studies on the brown marmorated stink bug *Halyomorpha halys* (Stål), a close relative of *N. viridula*, have proposed a dichromatic vision system comprising UV and green photoreceptors [23]. While direct molecular or histological evidence to confirm whether N. viridula possesses a dichromatic or trichromatic system remains lacking, field-based behavioral evaluations are essential to determine the functional significance of visible light, particularly blue light, in their attraction.

Although the attractiveness of monochromatic light across various wavelengths has been investigated in numerous species, few studies have examined the attractiveness of light sources combining multiple wavelengths. To address this gap, we evaluated the effects of combinations of UV and other visible light sources—including blue, green, orange, and red wavelengths—on the attractiveness of insect species under field conditions, primarily focusing on *Nezara* bugs. Specifically, this study aimed to determine which wavelength combinations significantly enhance the inherent attractiveness of UV light and to identify potential synergistic effects across the visible spectrum. These findings aim to provide a functional basis for developing wavelength-optimized light traps to support effective pest control strategies.

## 2. Materials and Methods

### 2.1. LED Traps

A commercially available portable light trap (Eco-Chu Trap, Konan Shisetsu Kanri, Okinawa, Japan) was used to modify the light source. Light sources with 42 LEDs were used in all experiments. Bullet-type UV-LED bulbs (NS395L-ERLO; 395 nm, 20 mA, Nitride Semiconductors, Tokushima, Japan), blue LED bulbs (OSB56P5111A; 470 nm, 10 mA, OptoSupply, Hong Kong, China), green LED bulbs (NEPG510S; 525 nm, 20 mA, Nichia, Tokushima, Japan), orange LED bulbs (OOS5OAA5111A; 605 nm, 20 mA, OptoSupply, Hong Kong, China) and red LED bulbs (OSR5CA5111A-WY; 625 nm, 18 mA, OptoSupply, Hong Kong, China) were used. The LEDs were positioned vertically on a stainless-steel cylinder (diameter, 4.8 cm; height, 20 cm). Light sources were organized into eight rows along the circumference. Each row consisted of five or six LEDs spaced at 3.6 cm intervals. Adjacent LEDs were arranged in a left-handed spiral (elevation angle, 63°; interval, approximately 2.0 cm). When utilizing both UV and other LEDs, the arrangement involved alternating the LEDs in a linear configuration. The cylinder containing LEDs was encased in a transparent acrylic cylinder (diameter, 9.8 cm; height, 20 cm).

**Figure 1:**
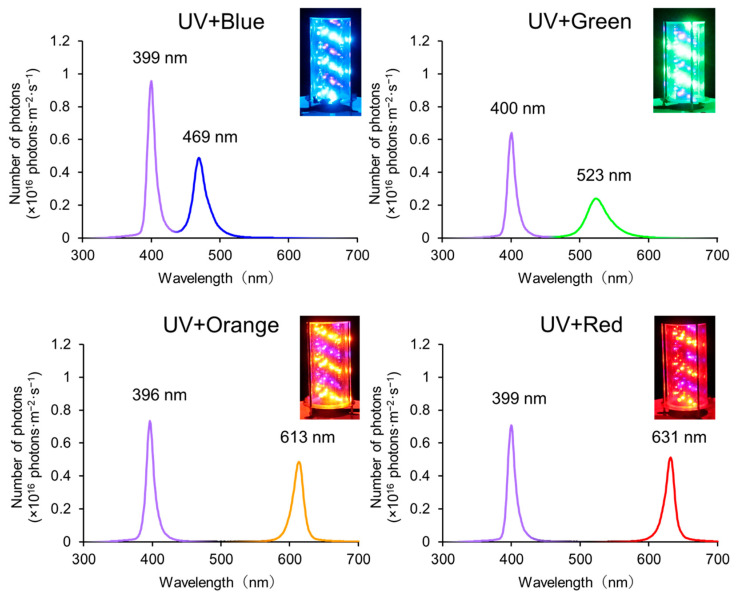
Emission spectra of UV-based multichromatic lights. The light intensity was measured using a high-speed spectrometer (HSU-100S). An attached sensor fiber was placed 50 cm in front of the light source.

The light source was mounted on a funnel (diameter, 31 cm; height, 24 cm), with the lower part of the light source situated approximately 100 cm above the ground. The trap was designed such that insects attracted to the light source would fall through the funnel and into a cylindrical chamber (diameter, 23 cm; height, 20 cm) positioned directly beneath it. A dimethyl-dichloro-vinyl-phosphate (DDVP) plate containing 10.7 g dichlorvos (Bapona Earth Chemical, Tokyo, Japan) was placed inside the chamber to kill the captured insects. The legs of the traps were anchored to the ground with steel stakes. The lights were activated at 18:00 and deactivated at 6:00 the following day. The lights were powered by rechargeable car batteries (N-40B19R/SB; DC 12V, 28 Ah, Panasonic, Osaka, Japan). The configuration of the light source used in this study is shown in Figure 1. For a photograph of the entire trap assembly, see Endo et al. [20].

### 2.2. Emission Spectra of Combined Light

The spectral intensity of combinations of UV and other visible light was measured using a high-speed spectrometer (HSU-100S; Asahi Spectra, Tokyo, Japan) in a controlled dark environment. An attached sensor fiber was placed 50 cm in front of the light source. The measurement was performed five times, with the light source rotated for each instance to minimize angular effects, and the average was taken as the representative value. The emission spectra of the UV-based multichromatic lights are shown in Figure 1. The UV- and blue-LED emission spectra exhibited single peaks at wavelengths of 399 and 469 nm, respectively. The calculated light intensities for the UV (330–425 nm) and blue (426–530 nm) regions were 1.55 × 10^17^ and 1.41 × 10^17^ photons m^−2^ s^−1^, respectively. The UV and green LED emission spectra exhibited single peaks at wavelengths of 400 and 523 nm, respectively. The calculated light intensities for the UV (330–450 nm) and green (451–600 nm) regions were 1.07 × 10^17^ and 1.05 × 10^17^ photons m^−2^ s^−1^, respectively. The UV- and orange-LED emission spectra exhibited single peaks at wavelengths of 396 and 613 nm, respectively. The calculated light intensities for the UV (330–450 nm) and orange (550–700 nm) regions were 1.23 × 10^17^ and 1.08 × 10^17^ photons m^−2^ s^−1^, respectively. The UV- and red-LED emission spectra exhibit single peaks at wavelengths of 399 and 631 nm, respectively. The calculated light intensities for the UV (330–450 nm) and red (550–700 nm) regions were 1.16 × 10^17^ and 1.07 × 10^17^ photons m^−2^ s^−1^, respectively. The light intensities of the visible LEDs ranged from 87.7 to 98.0% of UV, demonstrating near equivalence to UV levels.

### 2.3. Field Evaluation of Attractiveness to Light Sources

Field experiments were conducted at two locations in Japan: (1) Central Region Agricultural Research Center (CARC), Hokuriku Research Station (37°07′00″ N, 138°16′23″ E) in Niigata; (2) Yamaguchi Prefectural Agriculture & Forestry General Technology Center (YPATC) (34°09′37″ N, 131°29′47″ E) in Yamaguchi. The distribution of *Nezara* spp. differs across various regions in Japan. Only *N. antennata* has been found at Niigata. Both *N. antennata* and *N. viridula* have been identified in Yamaguchi; however, only *N. viridula* was caught at this location in our experiments.

#### 2.3.1. Experiment 1: Attractiveness of UV-Based Multichromatic Lights

Field experiments assessing the attractiveness of multichromatic lights were conducted from 30 July to 7 September 2019, near a soybean field at the CARC in Niigata and from 26 August to 16 October 2024, near a soybean field at the YPATC in Yamaguchi.

Light sources comprised multichromatic light traps featuring 21 UV-LEDs paired with 21 blue-LEDs, 21 green-LEDs, 21 orange-LEDs, 21 red-LEDs. A monochromatic light trap with 42 UV-LEDs was used as the positive control. The five LED traps were randomly positioned within the soybean field, with a minimum spacing of 20 m between each trap. Although insects other than *Nezara* bugs were captured in the light traps, the funnel-type traps used for soybean pest monitoring were designed to target large insects (>1 cm). Therefore, we restricted our counts to insects that met these conditions. Statistical analyses were performed on species with a total capture exceeding 50 individuals across the five traps. The species included *Nezara* bugs, the heteropteran bug *Piezodorus hybneri* (Gmelin), the lepidopteran moth *Pleuroptya ruralis* (Scopoli), and the coleopteran beetles *Anomala albopilosa* (Hope) and *A. rufocuprea* Motschulsky. The insects captured in the traps were counted for each species every 3 days in Niigata and every 3–4 days in Yamaguchi. The traps were randomly replaced on a weekly basis to minimize the effect of trap location.

#### 2.3.2. Experiment 2: Attractiveness of Monochromatic and Combined UV and Blue Light

Field experiments were conducted to evaluate the attractiveness of combinations of UV and blue LEDs from 20 July to 29 August 2022, near a soybean field at the CARC in Niigata and from 25 July to 3 October 2023, near a soybean field at the YPATC in Yamaguchi. Light traps with 42 UV-LEDs, 42 blue-LEDs, and a combination of 21 UV-LEDs and 21 blue-LEDs were used as light sources. The three LED traps were randomly placed within the soybean field, with a minimum spacing of 20 m between each trap. The number of *Nezara* bugs captured in the traps was counted every 2–3 days in Niigata and every 7 days in Yamaguchi. Traps were randomly replaced on a weekly basis.

### 2.4. Statistical Analysis

In Experiment 1, the attractiveness of each multichromatic light source was compared with that of UV light using the Wilcoxon matched-pair signed-rank test. In Experiment 2, the effect of light source on trap catches was analyzed using the Friedman test, followed by the Wilcoxon signed-rank test with Bonferroni correction for multiple comparisons. To account for the repeated sampling of the same traps over time, we paired the data by each specific sampling occasion (i.e., each 3–4 day interval). This approach effectively controls for temporal fluctuations in insect activity and environmental conditions (e.g., temperature, moon phase) by focusing on the relative performance of traps within the same time-window. Additionally, the weekly randomization of trap locations was employed to minimize spatial bias, effectively treating the location effect as a controlled variable. Statistical analyses were performed using R version 4.5.1 [24].

## 3. Results

### 3.1. Attractiveness of UV-Based Multichromatic Light Sources to Nezara Bugs

**Figure 2:**
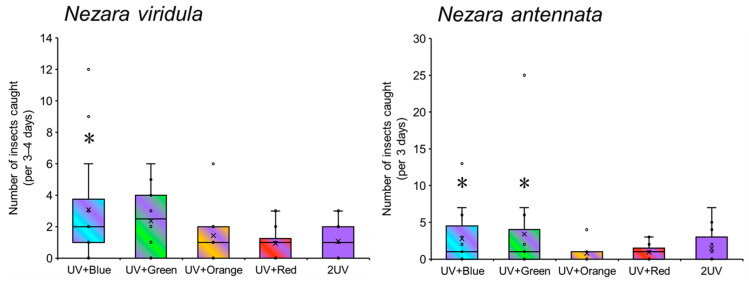
Attractiveness of UV-based multichromatic light sources to *Nezara viridula* (**left**) and *N. antennata* (**right**). Boxplots illustrate the median value (horizontal line), mean value (cross mark), interquartile range (boxed area), maximum and minimum values (vertical bar), and outlier value (circle). Asterisks indicate significant differences (*p* < 0.05) between monochromatic UV light and each UV-based multichromatic light (Wilcoxon signed-rank test).

The effectiveness of the UV-based multichromatic light traps in attracting *Nezara* bugs differed based on the specific light combinations employed (Figure 2, left). Traps combining UV and blue light resulted in a significantly greater number of *N. viridula* captured than the monochromatic UV light trap (Wilcoxon signed-rank test; *p* < 0.05) (Figure 2 left). Although the UV and green light trap captured more than twice the number of bugs as the monochromatic UV light trap, this difference represented only a marginal trend and was not statistically significant (Wilcoxon signed-rank test, *p* = 0.070). The attractiveness of the traps combining UV light and either orange or red light was similar to that of the monochromatic UV light trap (Wilcoxon signed-rank test; *p* > 0.05). The response of *N. antennata* to the UV-based multichromatic light traps mirrored that of *N. viridula* in overall pattern. Specifically, the UV and blue or green light traps captured significantly more *N. antennata* than the monochromatic UV light trap (Wilcoxon signed-rank test, *p* < 0.05) (Figure 2, right). However, the attractiveness of the traps combining UV and orange or red light was not significantly different from that of the monochromatic UV light trap (Wilcoxon signed-rank test, *p* > 0.05). The number captured was approximately half that of the monochromatic UV light trap, indicating that these color combinations had no positive effect.

### 3.2. Attractiveness of UV-Based Multichromatic Light Sources to Other Species

**Figure 3:**
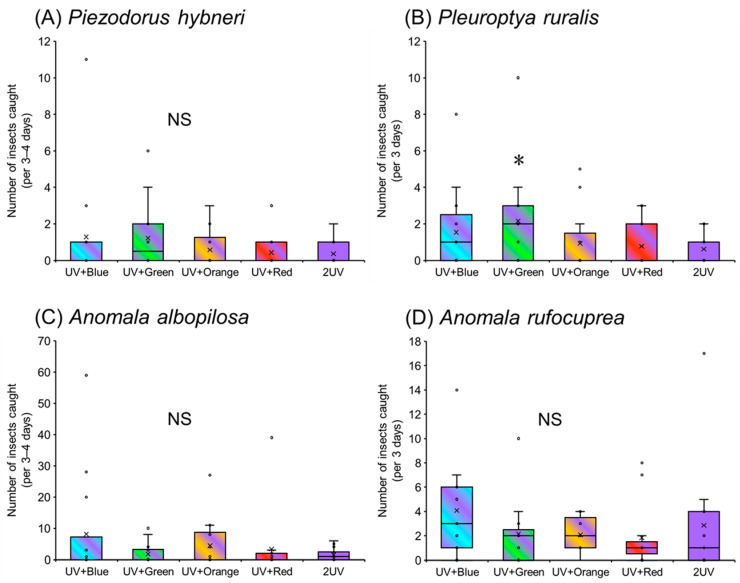
Attractiveness of UV-based multichromatic light sources to insect species other than *Nezara* bugs. Boxplots illustrate the median value (horizontal line), mean value (cross mark), interquartile range (boxed area), maximum and minimum values (vertical bar), and outlier value (circle). Asterisks indicate significant differences (*p* < 0.05) between monochromatic UV light and each UV-based multichromatic light (Wilcoxon signed-rank test). NS; not significant (*p* > 0.05).

Heteropteran bugs *P. hybneri* were captured in the traps combining UV and blue or green light at more than three times the rate compared with those captured in the monochromatic UV light trap; however, no significant differences were observed between them (Wilcoxon signed-rank test; *p* > 0.05) (Figure 3A). Many lepidopteran moths *P. ruralis* were caught in both the UV paired with blue or green light traps (Figure 3B). Notably, traps combining UV and green light captured more than 3.5 times the number of moths than the monochromatic UV light trap (Wilcoxon signed-rank test; *p* < 0.05). Coleopteran beetles *A. albopilosa* and *A. rufocuprea* were predominantly captured in traps combining UV and blue light; however, no significant differences were observed between any of the combined and monochromatic UV light traps (Wilcoxon signed-rank test; *p* > 0.05) (Figure 3C,D).

### 3.3. Attractiveness of Monochromatic- and Combined-UV and Blue Light

**Figure 4:**
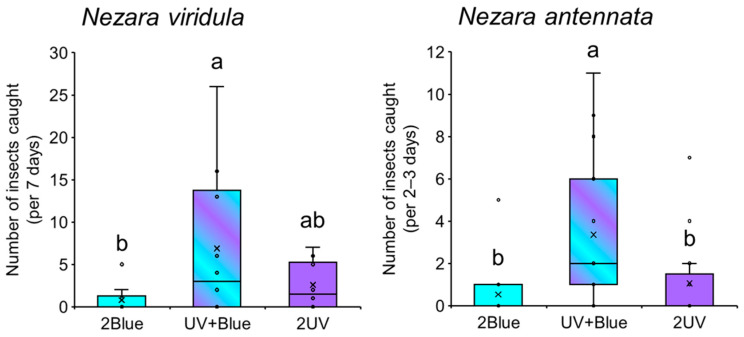
Attractiveness of monochromatic- and combined UV and blue light to *Nezara viridula* (**left**) and *N. antennata* (**right**). Boxplots illustrate the median value (horizontal line), mean value (cross mark), interquartile range (boxed area), maximum and minimum values (vertical bar), and outlier value (circle). Different letters above the bars indicate significant differences (*p* < 0.05) as determined by the Wilcoxon signed-rank test with Bonferroni correction.

The trap combining UV and blue light captured 8.6 times more *N. viridula* than that captured in the monochromatic blue light trap (Wilcoxon signed-rank test with Bonferroni correction; *p* < 0.05) (Figure 4, left). The monochromatic blue light trap captured fewer bugs than the monochromatic UV light trap, although this difference was not statistically significant (Wilcoxon signed-rank test with Bonferroni correction, *p* > 0.05). This indicates limited attractiveness. Similar results were observed for *N. antennata*, with the majority captured in the combined light trap, which was 6.3 times more effective than the monochromatic blue light trap and 3.2 times more effective than the monochromatic UV light trap (Figure 4, right). These results suggest that the trap combining UV and blue light enhances attractiveness to *Nezara* bugs in a synergistic rather than additive manner.

## 4. Discussion

Our field bioassays demonstrated that *Nezara* bugs were more strongly attracted to a combination of UV and blue light than to monochromatic UV light. This effect is comparable to that observed with the combination of UV and green light, which has been confirmed to exert a synergistic effect [20]. In contrast, the attractiveness of orange and red light paired with UV light was equivalent to or less than that of monochromatic UV light, thereby indicating that these color combinations do not enhance attraction. Furthermore, our field bioassays indicated that blue light alone tended to be less attractive to *Nezara* bugs than UV light. This is consistent with laboratory multiple-choice experiments using LEDs of various wavelengths, which revealed that no specific wavelength-dependent behaviors have been observed in *N. viridula*; instead, there is a clear hierarchy of attraction where UV is the most attractive, followed by blue, green, and then orange, with the attractiveness of UV being overwhelmingly dominant [19]. These findings suggest that while blue light is not highly attractive on its own, it enhances the attractiveness of UV light when combined with it.

Many insects are strongly attracted to UV light [16,17], while a strong attraction to blue light has also been reported in several species. Most lepidopteran moths are strongly attracted to short-wavelength light, particularly within the visible spectrum, where they are strongly attracted to blue light [25]. In the fall armyworm *Spodoptera frugiperda* (JE Smith) (Lepidoptera: Noctuidae), adult males are more strongly attracted to blue light than to UV light [26]. The diurnal western flower thrip *Frankliniella occidentalis* (Pergande) (Thysanoptera: Thripidae) is more strongly attracted to blue light than to other light sources, including UV light [27,28]. The brown marmorated stink bug *Halyomorpha halys* (Stål) (Hemiptera: Pentatomidae) exhibits a strong attraction to UV and blue light [29]. Furthermore, laboratory choice assays have indicated a preference for a combination of UV and blue light over UV light alone [30]. Conversely, field evaluations that incorporated pheromones did not reveal any advantage for insects when exposed to a combination of UV and blue light than to UV light alone [30]. This discrepancy may arise from the presence of pheromones or differences in experimental scales. Our study, conducted in a natural field setting without chemical lures, clearly demonstrates that the addition of blue light significantly enhances the attractiveness of UV light. To the best of our knowledge, this is the first field-based evidence of a clear synergistic increase in attractiveness resulting from combining blue light with other wavelengths in such agricultural pest species.

The precise mechanism by which the combination of various wavelengths enhances attractiveness remains unclear. However, distinct wavelengths elicit specific behaviors in certain diurnal hemipterans. UV radiation triggers migratory behaviors associated with flight initiation, migration, and dispersal [31,32,33], whereas green-yellow light promotes settling on host plants [31,32,33,34]. Furthermore, an antagonistic relationship between blue and green light has been observed in *T. vaporariorum* [33,35] and *F. occidentalis* [28]. Although such wavelength-specific behaviors have not been confirmed in *N. viridula*, the synergistic effects observed in our study suggest that *Nezara* bugs may exhibit complex behavioral responses to certain wavelength combinations, potentially improving capture efficiency. Further investigation is required to elucidate the mechanisms by which blue and green light, each displaying limited attractiveness on its own, enhance the attractiveness of UV light.

The attractiveness of combined-color light sources differs across species. In Coleopteran beetles, specifically *A. albopilosa* and *A. rufocuprea*, no significant difference in attraction is observed between UV-based multichromatic and UV light, indicating that UV photoreceptors predominantly influence light attraction in these species. In addition to *Nezara* bugs, the bean webworm *P. ruralis* (Lepidoptera) exhibits significantly higher attraction to a combination of UV and green light than to monochromatic UV light. The attractiveness of combined-color light to lepidopteran insects has also been studied in the oriental armyworm *Mythimna separata* (Walker), which demonstrates a stronger attraction to monochromatic green light (520 nm) than to a combination of two or three LEDs with varying wavelengths (400, 450, and 520 nm) [36]. Although the attractiveness of monochromatic green light remains unexplored, the enhanced attraction to combined-color light observed in *P. ruralis* suggests that a similar effect may be present in related moth species and in nocturnal insects of other orders.

Light traps that exploit insect-positive phototaxis have been employed to monitor numerous pest species [10,37]. Light sources attract not only pests but also their natural enemies and non-target insects [38,39,40], necessitating the development of light sources that effectively attract target species. The observed variations in attractiveness across different light combinations highlight the importance of spectral composition in insect attraction and may be leveraged to develop more effective and species-specific light traps. Furthermore, examining the mechanisms underlying insect attraction to specific light wavelengths and combinations may provide valuable insights into insect phototactic behavior and visual perception.

## Data Availability

The original contributions presented in this study are included in the article/Supplementary Material. Further inquiries can be directed to the corresponding author.

## Supplementary Materials

The following supporting information can be downloaded at: https://www.mdpi.com/article/10.3390/insects17030270/s1, Table S1: Raw data of the emission spectra of UV-based multichromatic lights; Table S2: Raw captured data of insects attracted to UV-based multichromatic lights; Table S3: Raw captured data of insects attracted to monochromatic UV, blue lights and combined UV and green light.

## Abbreviations

The following abbreviations are used in this manuscript:

CARC	Central Region Agricultural Research Center
DDVP	Dimethyl-dichloro-vinyl-phosphate (insecticidal plate containing dichlorvos)
ERG	Electroretinography
LED	Light-emitting diode
NS	Not significant
UV	Ultraviolet
YPATC	Yamaguchi Prefectural Agriculture & Forestry General Technology Center
