# Supplementary material for: Attractiveness of Green Stink Bugs Nezara spp. to Ultraviolet-Based Multichromatic Light Traps: Synergistic Effects of Ultraviolet and Blue Light
Source: Insects. 2026 Mar 3;17(3):270. doi: 10.3390/insects17030270 (PMC13026388; doi:10.3390/insects17030270)
Supplement: Supplementary file 1 [file insects-17-00270-s001.zip › Table S1.pdf]

Supplementary Table S1. Raw data of the emission spectra of UV-based multichromatic lights.

Wavelength [nm]	Light source (photons／(㎡・sec)			
	UV+Blue	UV+Green	UV+Orange	UV+Red
250	1.60108E+12	1.25566E+12	3.81892E+11	7.06455E+11
251	3.13185E+12	5.2993E+11	1.39079E+12	1.42888E+12
252	2.98766E+12	1.02644E+12	1.60958E+12	1.91867E+12
253	9.91794E+11	6.91493E+11	1.38805E+12	3.03069E+12
254	3.73428E+11	7.15288E+11	9.44805E+11	2.36206E+12
255	1.01008E+12	8.39315E+11	1.00629E+12	2.04765E+12
256	2.11287E+12	9.29973E+11	2.15617E+12	1.95471E+12
257	2.18603E+12	9.83308E+11	3.94679E+12	2.7068E+11
258	4.8247E+11	1.53942E+12	3.0947E+12	5.13933E+11
259	3.36328E+12	6.61192E+11	9.29289E+11	6.13378E+11
260	2.21559E+12	1.29041E+12	1.52121E+12	2.45574E+12
261	1.41219E+12	2.06725E+12	2.76526E+12	2.62008E+12
262	3.46437E+12	1.44603E+12	2.48528E+12	8.62485E+11
263	3.67413E+12	4.90642E+11	1.82103E+12	1.27437E+12
264	2.5099E+12	1.32134E+12	2.28101E+12	2.79819E+12
265	3.18388E+12	1.55054E+12	1.88584E+12	1.25196E+12
266	4.24615E+12	1.22452E+12	2.60285E+12	1.97339E+12
267	5.11525E+12	1.56151E+12	2.6913E+12	2.35034E+12
268	4.99049E+12	2.09774E+12	1.76893E+12	1.18217E+12
269	2.26437E+12	1.3151E+11	3.01561E+12	3.32461E+12
270	2.70917E+12	7.34939E+11	4.20018E+12	1.7271E+12
271	3.85304E+12	2.03187E+12	3.40859E+12	1.19829E+12
272	2.64564E+12	2.00383E+12	2.73368E+12	1.92107E+12
273	2.26485E+12	1.88323E+12	3.09637E+12	2.10264E+12
274	3.91182E+12	2.49426E+12	3.10032E+12	1.04687E+12
275	1.94301E+12	1.54212E+12	1.26499E+12	2.05791E+12
276	2.66355E+12	1.73227E+12	2.65835E+12	1.07636E+12
277	3.23619E+12	2.82632E+12	2.70592E+12	1.46148E+12
278	2.3649E+12	2.29404E+12	2.31966E+12	2.75196E+12
279	1.74883E+12	9.97658E+11	2.11536E+12	3.04975E+12
280	2.51535E+12	9.26772E+11	2.1884E+12	2.07901E+12
281	1.50334E+12	1.60038E+12	2.44791E+12	2.0894E+12
282	2.33892E+12	2.21655E+12	2.27052E+12	1.67442E+12
283	3.08166E+12	2.39346E+12	2.27784E+12	3.07324E+12
284	2.26199E+12	1.43104E+12	2.70259E+12	3.33687E+12
285	1.61806E+12	9.54877E+11	2.80063E+12	2.2155E+12
286	3.02271E+12	2.26803E+12	2.25007E+12	2.7144E+12
287	2.33788E+12	1.53473E+12	3.14205E+12	4.14555E+12
288	3.5418E+12	2.74132E+12	3.44634E+12	4.30516E+12
289	3.27138E+12	2.68509E+12	3.93188E+12	3.34571E+12

290	3.76319E+12	1.86003E+12	4.34573E+12	2.74369E+12
291	5.21967E+12	1.84375E+12	3.90564E+12	3.12676E+12
292	4.16255E+12	2.76624E+12	2.7004E+12	3.32147E+12
293	6.28614E+12	3.36909E+12	3.07395E+12	1.66753E+12
294	5.64952E+12	2.49303E+12	3.10305E+12	1.72289E+12
295	3.6743E+12	1.9685E+12	5.0833E+12	2.94564E+12
296	3.65219E+12	2.5036E+12	6.40914E+12	3.69563E+12
297	5.25268E+12	3.29498E+12	5.71794E+12	3.12464E+12
298	4.3953E+12	3.52713E+12	6.96979E+12	3.23103E+12
299	5.47399E+12	4.67916E+12	5.22034E+12	2.43097E+12
300	6.74854E+12	4.40388E+12	5.55672E+12	3.04704E+12
301	5.76186E+12	4.06495E+12	6.77562E+12	3.58614E+12
302	4.88111E+12	4.32412E+12	7.25324E+12	4.15378E+12
303	6.69024E+12	4.55703E+12	6.9226E+12	5.01078E+12
304	6.98842E+12	4.57216E+12	6.41406E+12	3.49383E+12
305	4.45399E+12	4.29452E+12	7.26026E+12	4.31323E+12
306	6.06807E+12	3.44139E+12	6.51325E+12	4.76282E+12
307	7.66719E+12	3.5061E+12	6.10078E+12	4.67677E+12
308	7.50521E+12	4.77589E+12	6.8459E+12	4.8817E+12
309	8.84933E+12	5.73048E+12	7.07401E+12	5.48517E+12
310	8.65488E+12	5.49343E+12	7.34511E+12	5.64769E+12
311	8.2969E+12	5.33697E+12	7.31087E+12	5.59813E+12
312	8.65182E+12	5.42806E+12	7.87305E+12	6.22113E+12
313	7.49125E+12	4.89779E+12	7.57604E+12	6.19718E+12
314	6.05524E+12	4.42588E+12	6.94314E+12	5.15768E+12
315	8.97471E+12	5.8848E+12	8.76672E+12	5.03306E+12
316	8.26049E+12	5.68254E+12	7.57476E+12	5.40194E+12
317	8.7941E+12	5.6266E+12	8.05092E+12	5.3671E+12
318	9.30582E+12	5.65601E+12	8.08188E+12	5.51457E+12
319	9.64343E+12	5.8574E+12	8.15764E+12	5.9531E+12
320	1.03538E+13	6.2978E+12	9.18888E+12	6.41778E+12
321	1.0181E+13	6.46952E+12	8.38275E+12	7.09053E+12
322	9.74038E+12	7.11468E+12	9.75575E+12	8.01403E+12
323	1.06051E+13	6.98781E+12	1.13899E+13	8.20287E+12
324	1.19963E+13	7.37705E+12	1.17173E+13	8.5144E+12
325	1.27803E+13	8.4281E+12	1.16248E+13	9.24364E+12
326	1.31503E+13	8.82319E+12	1.2857E+13	9.898E+12
327	1.48084E+13	8.8991E+12	1.41339E+13	1.10924E+13
328	1.65478E+13	9.76719E+12	1.51816E+13	1.18418E+13
329	1.85491E+13	1.11128E+13	1.6145E+13	1.16074E+13
330	1.97516E+13	1.24031E+13	1.77927E+13	1.25494E+13
331	2.03135E+13	1.31265E+13	1.99169E+13	1.47221E+13
332	2.20812E+13	1.31841E+13	2.11948E+13	1.49989E+13
333	2.35499E+13	1.38511E+13	2.21724E+13	1.54576E+13
334	2.55167E+13	1.54598E+13	2.4406E+13	1.75635E+13

335	2.80688E+13	1.66502E+13	2.66306E+13	1.8625E+13
336	3.06512E+13	1.77411E+13	2.81649E+13	1.96873E+13
337	3.301E+13	1.9547E+13	2.99918E+13	2.24584E+13
338	3.5825E+13	2.10247E+13	3.34878E+13	2.38236E+13
339	3.79813E+13	2.23945E+13	3.49999E+13	2.48195E+13
340	4.15811E+13	2.47339E+13	3.78018E+13	2.66164E+13
341	4.49674E+13	2.68868E+13	4.01838E+13	2.92951E+13
342	4.68258E+13	2.8332E+13	4.21352E+13	3.20126E+13
343	4.83405E+13	2.99584E+13	4.55601E+13	3.36453E+13
344	5.15227E+13	3.19198E+13	4.68397E+13	3.36218E+13
345	5.35814E+13	3.33372E+13	4.99403E+13	3.59417E+13
346	5.68914E+13	3.47425E+13	5.36703E+13	3.8687E+13
347	6.11891E+13	3.6761E+13	5.67933E+13	4.09489E+13
348	6.53535E+13	3.895E+13	5.90714E+13	4.30088E+13
349	6.96684E+13	4.0316E+13	6.12132E+13	4.49484E+13
350	7.2019E+13	4.38499E+13	6.52583E+13	4.63782E+13
351	7.4102E+13	4.51621E+13	6.89087E+13	4.89275E+13
352	7.63538E+13	4.69284E+13	7.14979E+13	5.20516E+13
353	7.985E+13	4.95521E+13	7.42509E+13	5.51216E+13
354	8.43655E+13	5.09595E+13	7.79986E+13	5.77625E+13
355	8.60792E+13	5.22731E+13	8.0744E+13	5.92289E+13
356	9.14901E+13	5.57768E+13	8.53447E+13	6.14746E+13
357	9.75173E+13	5.77335E+13	8.91501E+13	6.45044E+13
358	1.01109E+14	5.94841E+13	9.18157E+13	6.64356E+13
359	1.03489E+14	6.21758E+13	9.46047E+13	6.84336E+13
360	1.08213E+14	6.39811E+13	9.83025E+13	7.34413E+13
361	1.11401E+14	6.61048E+13	1.01641E+14	7.40314E+13
362	1.1495E+14	6.89182E+13	1.06397E+14	7.6826E+13
363	1.19864E+14	7.08271E+13	1.09987E+14	7.97163E+13
364	1.23953E+14	7.24768E+13	1.12658E+14	8.18494E+13
365	1.26341E+14	7.50949E+13	1.15962E+14	8.42876E+13
366	1.31489E+14	7.75418E+13	1.18919E+14	8.70634E+13
367	1.36416E+14	8.10903E+13	1.23887E+14	9.1022E+13
368	1.41723E+14	8.43558E+13	1.29914E+14	9.47758E+13
369	1.46056E+14	8.73751E+13	1.34837E+14	9.76308E+13
370	1.50395E+14	9.04024E+13	1.39271E+14	1.00261E+14
371	1.57079E+14	9.33699E+13	1.45685E+14	1.04044E+14
372	1.62162E+14	9.68261E+13	1.51889E+14	1.0933E+14
373	1.70626E+14	1.01133E+14	1.62782E+14	1.1355E+14
374	1.78665E+14	1.05668E+14	1.76209E+14	1.17911E+14
375	1.86001E+14	1.10823E+14	1.93104E+14	1.24067E+14
376	1.93797E+14	1.16399E+14	2.15092E+14	1.31091E+14
377	2.00803E+14	1.20819E+14	2.41829E+14	1.3569E+14
378	2.1325E+14	1.28183E+14	2.84654E+14	1.42516E+14
379	2.24243E+14	1.34969E+14	3.39676E+14	1.49811E+14

380	2.37527E+14	1.43182E+14	4.12204E+14	1.58843E+14
381	2.55265E+14	1.54039E+14	5.06417E+14	1.69996E+14
382	2.7731E+14	1.67682E+14	6.25263E+14	1.84204E+14
383	3.1343E+14	1.89683E+14	7.81598E+14	2.09432E+14
384	3.70732E+14	2.25008E+14	9.80722E+14	2.51041E+14
385	4.61108E+14	2.80034E+14	1.23087E+15	3.1619E+14
386	6.00841E+14	3.63937E+14	1.54157E+15	4.1646E+14
387	8.07681E+14	4.8691E+14	1.91971E+15	5.66342E+14
388	1.10005E+15	6.59985E+14	2.37235E+15	7.81919E+14
389	1.50601E+15	9.03857E+14	2.93452E+15	1.08594E+15
390	2.05221E+15	1.23059E+15	3.61022E+15	1.4944E+15
391	2.74734E+15	1.64961E+15	4.37847E+15	2.01999E+15
392	3.58367E+15	2.15955E+15	5.18809E+15	2.65828E+15
393	4.55395E+15	2.75817E+15	5.97926E+15	3.39819E+15
394	5.67045E+15	3.46222E+15	6.71653E+15	4.24759E+15
395	6.76823E+15	4.1769E+15	7.17721E+15	5.08191E+15
396	7.79641E+15	4.87451E+15	7.34003E+15	5.85708E+15
397	8.61379E+15	5.46843E+15	7.16241E+15	6.46108E+15
398	9.14937E+15	5.91061E+15	6.69291E+15	6.83839E+15
399	9.50252E+15	6.25335E+15	6.13712E+15	7.06668E+15
**400**	9.55778E+15	6.41815E+15	5.51448E+15	7.06263E+15
401	9.07788E+15	6.21784E+15	4.77444E+15	6.66355E+15
402	8.25063E+15	5.75706E+15	4.05533E+15	6.01796E+15
403	7.30972E+15	5.18126E+15	3.46239E+15	5.30627E+15
404	6.35784E+15	4.55832E+15	2.98601E+15	4.60533E+15
405	5.48519E+15	3.95556E+15	2.6046E+15	3.96781E+15
406	4.74109E+15	3.42232E+15	2.29631E+15	3.42923E+15
407	4.125E+15	2.96839E+15	2.03438E+15	2.98545E+15
408	3.61989E+15	2.59215E+15	1.80275E+15	2.62121E+15
409	3.20242E+15	2.28348E+15	1.59355E+15	2.31782E+15
410	2.84655E+15	2.02298E+15	1.40084E+15	2.0553E+15
411	2.53468E+15	1.79654E+15	1.22739E+15	1.82137E+15
412	2.25242E+15	1.59399E+15	1.07453E+15	1.60938E+15
413	1.99395E+15	1.4094E+15	9.40946E+14	1.41529E+15
414	1.76011E+15	1.24101E+15	8.26475E+14	1.2401E+15
415	1.5542E+15	1.08998E+15	7.3192E+14	1.08709E+15
416	1.37755E+15	9.58527E+14	6.54262E+14	9.54295E+14
417	1.22844E+15	8.45378E+14	5.88725E+14	8.43231E+14
418	1.1037E+15	7.49716E+14	5.33486E+14	7.5019E+14
419	9.97953E+14	6.68823E+14	4.85324E+14	6.71656E+14
420	9.07247E+14	5.99633E+14	4.41319E+14	6.05203E+14
421	8.31731E+14	5.40868E+14	4.01523E+14	5.48313E+14
422	7.67766E+14	4.91068E+14	3.64826E+14	4.96257E+14
423	7.08154E+14	4.47276E+14	3.30932E+14	4.50695E+14
424	6.53851E+14	4.06314E+14	2.99817E+14	4.09617E+14

425	6.05838E+14	3.68184E+14	2.71348E+14	3.71226E+14
426	5.6165E+14	3.35063E+14	2.4552E+14	3.35961E+14
427	5.23345E+14	3.035E+14	2.23821E+14	3.02937E+14
428	4.89419E+14	2.75618E+14	2.02835E+14	2.73044E+14
429	4.60328E+14	2.50537E+14	1.85069E+14	2.4714E+14
430	4.35174E+14	2.28033E+14	1.70012E+14	2.24628E+14
431	4.13475E+14	2.08399E+14	1.55706E+14	2.0483E+14
432	3.98063E+14	1.90784E+14	1.44516E+14	1.88967E+14
433	3.8483E+14	1.76541E+14	1.33804E+14	1.73578E+14
434	3.76622E+14	1.63253E+14	1.23001E+14	1.58737E+14
435	3.72013E+14	1.51034E+14	1.13403E+14	1.45841E+14
436	3.69456E+14	1.40667E+14	1.05259E+14	1.34735E+14
437	3.70789E+14	1.3151E+14	9.69184E+13	1.23454E+14
438	3.76341E+14	1.22756E+14	9.03691E+13	1.14657E+14
439	3.85171E+14	1.15049E+14	8.43144E+13	1.06703E+14
440	3.97815E+14	1.08112E+14	7.80139E+13	9.83446E+13
441	4.14947E+14	1.01647E+14	7.1926E+13	9.00524E+13
442	4.36554E+14	9.59076E+13	6.69511E+13	8.33769E+13
443	4.61926E+14	9.17108E+13	6.26742E+13	7.8701E+13
444	4.9236E+14	8.7542E+13	5.83058E+13	7.35485E+13
445	5.30567E+14	8.37404E+13	5.52252E+13	6.81994E+13
446	5.75179E+14	8.04602E+13	5.2096E+13	6.32707E+13
447	6.24769E+14	7.76169E+13	4.80233E+13	5.89201E+13
448	6.82381E+14	7.5277E+13	4.5737E+13	5.49839E+13
449	7.47905E+14	7.30802E+13	4.30808E+13	5.32413E+13
450	8.23278E+14	7.14521E+13	4.04567E+13	5.03686E+13
451	9.10139E+14	6.9911E+13	3.90169E+13	4.61654E+13
452	1.01017E+15	6.8347E+13	3.76022E+13	4.28915E+13
453	1.12588E+15	6.74097E+13	3.3946E+13	4.19639E+13
454	1.26164E+15	6.7037E+13	3.29665E+13	3.81037E+13
455	1.4209E+15	6.66933E+13	3.08937E+13	3.61314E+13
456	1.60336E+15	6.62102E+13	2.95256E+13	3.4907E+13
457	1.81353E+15	6.60097E+13	2.83148E+13	3.33934E+13
458	2.05609E+15	6.64583E+13	2.57256E+13	3.17801E+13
459	2.3252E+15	6.691E+13	2.50823E+13	2.96745E+13
460	2.62079E+15	6.68986E+13	2.41106E+13	2.72644E+13
461	2.93692E+15	6.76136E+13	2.34274E+13	2.62536E+13
462	3.27266E+15	6.89438E+13	2.24558E+13	2.59425E+13
463	3.61936E+15	7.0353E+13	2.10129E+13	2.47939E+13
464	3.95282E+15	7.20317E+13	2.06338E+13	2.28575E+13
465	4.26064E+15	7.32422E+13	2.04964E+13	2.20872E+13
466	4.5225E+15	7.5121E+13	2.0129E+13	2.14346E+13
467	4.72276E+15	7.81043E+13	1.88674E+13	2.11968E+13
468	4.8494E+15	8.08252E+13	1.74244E+13	2.0615E+13
**469**	4.89162E+15	8.1817E+13	1.72424E+13	1.83019E+13

470	4.83556E+15	8.47586E+13	1.68267E+13	1.70689E+13
471	4.70631E+15	8.79305E+13	1.51944E+13	1.74135E+13
472	4.52002E+15	9.1885E+13	1.49077E+13	1.73592E+13
473	4.28914E+15	9.62825E+13	1.50209E+13	1.61853E+13
474	4.02641E+15	1.00386E+14	1.31379E+13	1.50279E+13
475	3.74888E+15	1.05132E+14	1.18364E+13	1.51725E+13
476	3.47621E+15	1.09642E+14	1.14468E+13	1.51592E+13
477	3.22411E+15	1.14997E+14	1.19549E+13	1.45421E+13
478	2.99343E+15	1.21293E+14	1.24449E+13	1.37935E+13
479	2.78082E+15	1.28555E+14	1.2171E+13	1.34253E+13
480	2.58936E+15	1.37366E+14	1.149E+13	1.3153E+13
481	2.41654E+15	1.4425E+14	1.10727E+13	1.20954E+13
482	2.26768E+15	1.52922E+14	1.00957E+13	1.07623E+13
483	2.13685E+15	1.62985E+14	9.93329E+12	1.02447E+13
484	2.01387E+15	1.74021E+14	1.04273E+13	1.04177E+13
485	1.89161E+15	1.87326E+14	9.6062E+12	1.00604E+13
486	1.76798E+15	2.01907E+14	9.91952E+12	1.0201E+13
487	1.64746E+15	2.16634E+14	9.48694E+12	9.08707E+12
488	1.5313E+15	2.33016E+14	8.84324E+12	7.81266E+12
489	1.41784E+15	2.51822E+14	8.84436E+12	7.6363E+12
490	1.30677E+15	2.72585E+14	9.22564E+12	8.45557E+12
491	1.20485E+15	2.95306E+14	8.03367E+12	8.52676E+12
492	1.10904E+15	3.20039E+14	8.38509E+12	7.5124E+12
493	1.01976E+15	3.48337E+14	8.84396E+12	7.71615E+12
494	9.39355E+14	3.79742E+14	8.31659E+12	8.42608E+12
495	8.68328E+14	4.13358E+14	7.37851E+12	7.86047E+12
496	8.02912E+14	4.50394E+14	7.65007E+12	6.76721E+12
497	7.4447E+14	4.92735E+14	7.10195E+12	6.50476E+12
498	6.92559E+14	5.39796E+14	7.68606E+12	6.99318E+12
499	6.45174E+14	5.89435E+14	8.83254E+12	7.37243E+12
500	6.0032E+14	6.41403E+14	8.813E+12	6.53414E+12
501	5.57924E+14	6.9845E+14	7.22305E+12	4.43596E+12
502	5.22361E+14	7.61181E+14	7.66558E+12	5.88142E+12
503	4.90233E+14	8.31362E+14	7.70499E+12	4.85722E+12
504	4.58789E+14	9.07875E+14	7.92292E+12	3.87962E+12
505	4.28754E+14	9.89345E+14	8.29119E+12	4.73005E+12
506	4.02434E+14	1.07564E+15	7.68619E+12	5.38895E+12
507	3.76697E+14	1.1676E+15	7.4441E+12	5.50206E+12
508	3.4988E+14	1.2635E+15	7.0643E+12	3.97901E+12
509	3.24396E+14	1.36043E+15	7.50766E+12	4.63499E+12
510	3.00219E+14	1.45797E+15	7.87845E+12	6.06378E+12
511	2.76951E+14	1.55719E+15	6.94826E+12	4.8577E+12
512	2.59505E+14	1.65701E+15	6.77056E+12	5.06233E+12
513	2.40739E+14	1.75645E+15	7.94134E+12	4.8436E+12
514	2.23651E+14	1.85582E+15	8.34455E+12	4.46809E+12

515	2.09003E+14	1.95389E+15	7.81199E+12	4.10639E+12
516	1.95162E+14	2.0471E+15	7.51608E+12	4.0696E+12
517	1.81344E+14	2.13076E+15	7.90191E+12	5.07043E+12
518	1.70881E+14	2.20579E+15	7.7706E+12	5.49217E+12
519	1.59794E+14	2.26845E+15	8.08547E+12	5.63478E+12
520	1.4921E+14	2.32106E+15	8.99467E+12	5.31019E+12
521	1.40965E+14	2.36422E+15	9.32634E+12	5.0527E+12
522	1.33695E+14	2.39237E+15	7.84396E+12	6.12837E+12
523	1.25878E+14	2.4041E+15	8.28378E+12	6.50417E+12
524	1.17101E+14	2.3999E+15	8.59456E+12	4.7086E+12
525	1.08843E+14	2.38358E+15	9.09024E+12	3.10988E+12
526	1.02208E+14	2.35479E+15	9.78701E+12	3.74073E+12
527	9.6732E+13	2.3112E+15	9.77248E+12	5.78978E+12
528	9.08642E+13	2.26466E+15	9.74848E+12	5.91227E+12
529	8.62458E+13	2.20867E+15	1.12925E+13	5.30962E+12
530	8.09746E+13	2.14139E+15	1.22994E+13	5.06448E+12
531	7.48461E+13	2.06573E+15	1.174E+13	5.39922E+12
532	6.96797E+13	1.98711E+15	1.10578E+13	5.94252E+12
533	6.60713E+13	1.90873E+15	1.27297E+13	6.92574E+12
534	6.24356E+13	1.83026E+15	1.32391E+13	5.75012E+12
535	5.90156E+13	1.75214E+15	1.27549E+13	5.84065E+12
536	5.51647E+13	1.67514E+15	1.29256E+13	6.51494E+12
537	5.07759E+13	1.60015E+15	1.42956E+13	5.43237E+12
538	4.83599E+13	1.52773E+15	1.44119E+13	4.9919E+12
539	4.60803E+13	1.45494E+15	1.43136E+13	6.42566E+12
540	4.3808E+13	1.38746E+15	1.51176E+13	8.14391E+12
541	4.1002E+13	1.32472E+15	1.53686E+13	8.91786E+12
542	3.80707E+13	1.26407E+15	1.46473E+13	8.31172E+12
543	3.69842E+13	1.20697E+15	1.60892E+13	6.75399E+12
544	3.5437E+13	1.15212E+15	1.70459E+13	8.17788E+12
545	3.30235E+13	1.09972E+15	1.80345E+13	8.26745E+12
546	3.07255E+13	1.04838E+15	1.87312E+13	9.05513E+12
547	2.92172E+13	9.97169E+14	1.92038E+13	1.10313E+13
548	2.80629E+13	9.47049E+14	2.03292E+13	9.53419E+12
549	2.8167E+13	8.98784E+14	2.05282E+13	9.30182E+12
550	2.56222E+13	8.52937E+14	2.10217E+13	1.11325E+13
551	2.25525E+13	8.10169E+14	2.267E+13	1.17366E+13
552	2.15679E+13	7.68874E+14	2.48306E+13	1.10189E+13
553	2.22215E+13	7.267E+14	2.58698E+13	1.25965E+13
554	2.20267E+13	6.88751E+14	2.59225E+13	1.25373E+13
555	2.09921E+13	6.50496E+14	2.71189E+13	1.30343E+13
556	1.82531E+13	6.14184E+14	2.86898E+13	1.39696E+13
557	1.57377E+13	5.79887E+14	3.05203E+13	1.43757E+13
558	1.62118E+13	5.45675E+14	3.32458E+13	1.42231E+13
559	1.6062E+13	5.16882E+14	3.47884E+13	1.67199E+13

560	1.46719E+13	4.89442E+14	3.74104E+13	1.767E+13
561	1.31131E+13	4.62948E+14	4.06659E+13	1.69708E+13
562	1.25082E+13	4.37388E+14	4.41406E+13	1.71333E+13
563	1.29693E+13	4.12915E+14	4.76998E+13	1.98981E+13
564	1.32561E+13	3.90953E+14	5.04242E+13	2.19889E+13
565	1.30825E+13	3.70795E+14	5.33872E+13	2.22432E+13
566	1.33872E+13	3.52065E+14	5.74443E+13	2.25186E+13
567	1.29148E+13	3.33679E+14	6.19845E+13	2.39735E+13
568	1.11277E+13	3.15311E+14	6.62706E+13	2.60706E+13
569	1.21885E+13	2.98976E+14	7.14464E+13	2.72085E+13
570	1.11183E+13	2.8115E+14	7.72437E+13	2.87129E+13
571	9.69234E+12	2.65534E+14	8.38075E+13	2.99351E+13
572	9.16559E+12	2.5174E+14	9.08913E+13	3.17109E+13
573	9.28467E+12	2.37697E+14	9.84911E+13	3.41933E+13
574	9.07968E+12	2.24518E+14	1.07412E+14	3.47905E+13
575	8.49633E+12	2.13349E+14	1.17193E+14	3.59043E+13
576	9.26029E+12	2.02574E+14	1.27877E+14	3.83832E+13
577	9.52505E+12	1.9135E+14	1.39951E+14	4.25451E+13
578	7.8158E+12	1.80535E+14	1.53685E+14	4.64743E+13
579	6.42872E+12	1.71948E+14	1.68845E+14	4.66336E+13
580	5.75099E+12	1.62821E+14	1.86612E+14	4.96431E+13
581	5.97718E+12	1.53972E+14	2.07615E+14	5.50805E+13
582	5.89912E+12	1.45509E+14	2.30687E+14	5.97206E+13
583	5.11964E+12	1.37415E+14	2.56146E+14	6.31617E+13
584	5.20201E+12	1.30188E+14	2.86814E+14	6.91612E+13
585	7.22232E+12	1.24647E+14	3.20968E+14	7.33261E+13
586	7.34956E+12	1.18238E+14	3.59272E+14	7.8776E+13
587	5.58083E+12	1.11589E+14	4.02304E+14	8.57618E+13
588	4.42249E+12	1.05989E+14	4.50605E+14	9.35631E+13
589	5.90305E+12	1.01228E+14	5.05013E+14	1.01661E+14
590	6.57605E+12	9.52861E+13	5.67489E+14	1.06967E+14
591	5.47833E+12	8.99102E+13	6.38932E+14	1.16159E+14
592	3.89266E+12	8.5278E+13	7.19482E+14	1.27692E+14
593	3.24376E+12	8.12738E+13	8.10279E+14	1.38657E+14
594	3.96328E+12	7.80036E+13	9.12681E+14	1.49419E+14
595	6.6863E+12	7.48176E+13	1.02262E+15	1.6397E+14
596	5.72434E+12	7.10425E+13	1.14474E+15	1.76086E+14
597	3.76989E+12	6.62447E+13	1.28159E+15	1.92575E+14
598	3.94986E+12	6.2317E+13	1.43235E+15	2.1341E+14
599	5.57223E+12	6.13549E+13	1.59475E+15	2.3084E+14
600	4.34011E+12	5.77768E+13	1.77063E+15	2.5321E+14
601	4.78007E+12	5.53058E+13	1.96715E+15	2.77613E+14
602	4.8042E+12	5.2903E+13	2.18655E+15	3.0376E+14
603	3.65229E+12	4.9858E+13	2.4277E+15	3.32905E+14
604	3.26128E+12	4.72982E+13	2.68808E+15	3.66069E+14

605	3.51439E+12	4.68573E+13	2.96574E+15	4.00631E+14
606	3.43406E+12	4.35499E+13	3.26593E+15	4.43239E+14
607	4.65291E+12	4.09047E+13	3.5773E+15	4.94206E+14
608	4.8258E+12	3.95362E+13	3.88626E+15	5.50008E+14
609	1.34316E+12	3.66801E+13	4.17993E+15	6.08037E+14
610	2.93798E+12	3.42339E+13	4.43983E+15	6.73455E+14
611	3.92937E+12	3.27529E+13	4.6618E+15	7.48062E+14
612	3.69428E+12	3.19014E+13	4.81452E+15	8.33745E+14
613	2.55304E+12	3.14146E+13	4.87288E+15	9.30942E+14
614	1.37756E+12	3.08711E+13	4.82545E+15	1.04091E+15
615	3.35343E+12	2.8607E+13	4.66376E+15	1.17023E+15
616	3.76768E+12	2.71846E+13	4.40095E+15	1.32015E+15
617	3.58322E+12	2.60857E+13	4.05227E+15	1.48943E+15
618	2.9431E+12	2.52004E+13	3.63522E+15	1.67181E+15
619	1.61621E+12	2.46976E+13	3.17666E+15	1.86128E+15
620	2.041E+12	2.30996E+13	2.72348E+15	2.0609E+15
621	3.12739E+12	2.18278E+13	2.30073E+15	2.26826E+15
622	2.45658E+12	2.09572E+13	1.92435E+15	2.4934E+15
623	1.46645E+12	2.01998E+13	1.60109E+15	2.7497E+15
624	2.56384E+12	1.95034E+13	1.33092E+15	3.04826E+15
625	6.42075E+11	1.94972E+13	1.11059E+15	3.39424E+15
626	2.50986E+12	1.81624E+13	9.25709E+14	3.76908E+15
627	3.95283E+12	1.65145E+13	7.72175E+14	4.15088E+15
628	3.00824E+12	1.62587E+13	6.49263E+14	4.51038E+15
629	1.7636E+12	1.71724E+13	5.53205E+14	4.81497E+15
630	2.13317E+12	1.48837E+13	4.71594E+14	5.03518E+15
631	1.51202E+12	1.5792E+13	4.06126E+14	5.12744E+15
632	1.38987E+12	1.49652E+13	3.51411E+14	5.07852E+15
633	2.85369E+12	1.29334E+13	3.0494E+14	4.886E+15
634	4.37931E+12	1.31485E+13	2.67737E+14	4.56257E+15
635	1.00258E+12	1.21249E+13	2.41068E+14	4.14584E+15
636	7.39977E+11	1.23096E+13	2.15258E+14	3.65697E+15
637	1.91451E+12	1.20765E+13	1.94334E+14	3.1274E+15
638	2.00649E+12	1.10122E+13	1.78453E+14	2.6006E+15
639	5.76475E+11	1.01543E+13	1.64579E+14	2.12234E+15
640	3.82056E+12	1.02434E+13	1.53962E+14	1.72576E+15
641	2.09412E+12	1.05735E+13	1.44081E+14	1.40882E+15
642	6.29227E+11	9.97642E+12	1.34868E+14	1.16895E+15
643	1.94155E+12	9.18159E+12	1.27364E+14	9.90233E+14
644	5.32556E+12	9.34844E+12	1.21789E+14	8.50944E+14
645	2.85046E+12	9.15566E+12	1.15575E+14	7.37928E+14
646	3.06502E+12	7.81345E+12	1.08716E+14	6.44267E+14
647	3.11912E+12	6.85922E+12	1.04965E+14	5.62955E+14
648	2.77201E+12	7.14255E+12	1.01468E+14	4.90037E+14
649	3.67145E+12	7.78444E+12	9.4667E+13	4.26179E+14

650	3.23196E+12	6.31077E+12	9.21616E+13	3.76865E+14
651	1.61622E+12	6.69598E+12	8.89132E+13	3.31034E+14
652	7.1014E+11	6.29954E+12	8.49053E+13	2.94393E+14
653	9.49419E+11	6.16289E+12	8.13729E+13	2.67281E+14
654	1.17674E+12	8.00431E+12	7.84448E+13	2.45028E+14
655	5.44136E+11	6.04737E+12	7.32115E+13	2.27641E+14
656	2.50415E+12	5.23931E+12	7.01519E+13	2.09158E+14
657	2.20825E+12	6.09746E+12	6.89022E+13	1.9507E+14
658	3.26159E+11	6.59923E+12	6.74341E+13	1.83982E+14
659	5.76685E+11	5.26364E+12	6.44793E+13	1.70757E+14
660	4.63039E+12	5.1139E+12	6.17997E+13	1.59727E+14
661	2.5292E+12	5.7893E+12	5.85727E+13	1.5165E+14
662	7.65843E+11	5.77691E+12	5.57838E+13	1.44878E+14
663	1.56572E+12	4.83839E+12	5.44319E+13	1.38502E+14
664	3.36909E+12	3.96879E+12	5.42857E+13	1.32105E+14
665	9.11377E+11	3.79897E+12	5.29094E+13	1.23446E+14
666	2.42536E+12	4.18768E+12	4.90755E+13	1.16419E+14
667	2.66167E+12	5.00587E+12	4.85343E+13	1.12983E+14
668	1.27257E+12	4.63901E+12	4.94759E+13	1.11321E+14
669	2.96218E+12	2.36871E+12	4.58882E+13	1.07837E+14
670	2.10609E+12	5.59642E+12	4.27404E+13	1.02016E+14
671	1.39527E+12	6.51035E+12	4.32815E+13	9.62917E+13
672	4.85976E+11	4.95017E+12	4.2746E+13	9.10909E+13
673	1.01184E+12	3.76918E+12	3.98181E+13	8.80576E+13
674	3.99008E+12	4.87679E+12	3.7512E+13	8.80358E+13
675	2.38915E+12	4.23881E+12	3.57119E+13	8.47903E+13
676	1.36741E+12	4.58841E+12	3.45355E+13	7.74688E+13
677	3.80073E+12	3.63806E+12	3.4615E+13	7.46224E+13
678	5.99764E+12	2.73099E+12	3.48332E+13	7.5744E+13
679	1.07453E+12	4.90934E+12	3.32447E+13	7.15115E+13
680	3.47851E+12	5.56606E+12	3.14051E+13	6.16615E+13
681	4.99459E+12	3.66958E+12	2.67757E+13	6.14691E+13
682	3.57714E+12	2.71018E+12	2.62791E+13	6.0305E+13
683	1.02161E+12	3.06327E+12	2.84814E+13	5.32417E+13
684	4.69334E+11	2.94466E+12	2.46384E+13	4.86683E+13
685	8.21362E+11	4.56491E+12	2.4256E+13	4.83546E+13
686	2.61585E+12	2.86825E+12	2.39362E+13	4.58439E+13
687	2.61213E+12	1.58688E+12	2.28036E+13	4.3669E+13
688	1.87918E+12	3.08117E+12	2.18864E+13	4.34112E+13
689	5.64584E+12	5.08661E+12	2.23954E+13	4.20961E+13
690	2.7616E+12	2.50886E+12	2.09634E+13	3.77786E+13
691	1.08873E+12	2.64385E+12	1.93863E+13	3.82192E+13
692	7.64481E+11	4.08655E+12	2.0292E+13	3.83911E+13
693	1.16459E+12	4.49664E+12	2.19728E+13	3.50898E+13
694	4.21309E+12	4.77066E+12	1.89412E+13	3.31942E+13

695	4.78915E+12	4.17984E+12	1.56928E+13	3.2105E+13
696	3.16321E+12	2.80783E+12	1.52918E+13	3.18028E+13
697	2.50037E+12	1.73537E+12	1.54189E+13	3.05762E+13
698	2.76717E+12	1.68477E+12	1.42437E+13	2.78621E+13
699	1.655E+12	3.95116E+12	1.38801E+13	2.55477E+13
700	1.17556E+12	3.98198E+12	1.45063E+13	2.63963E+13
701	2.75346E+12	4.55149E+12	1.39175E+13	2.59683E+13
702	3.54476E+12	5.59017E+12	1.35142E+13	2.4682E+13
703	1.56062E+12	5.25757E+12	1.3376E+13	2.42119E+13
704	1.00884E+12	2.3087E+12	9.61931E+12	2.38149E+13
705	7.76305E+11	2.93942E+12	9.0027E+12	2.34466E+13
706	4.62943E+11	4.01928E+12	9.5727E+12	2.07365E+13
707	1.81355E+12	3.40437E+12	1.04747E+13	1.99284E+13
708	4.03962E+12	1.9583E+12	1.07081E+13	2.27113E+13
709	9.30031E+11	3.06174E+12	7.59798E+12	2.01539E+13
710	4.64942E+11	4.52616E+12	7.48834E+12	1.85588E+13
711	2.84585E+12	3.25739E+12	7.89425E+12	1.8029E+13
712	4.06241E+12	1.34616E+12	9.45123E+12	1.85167E+13
713	1.0202E+12	2.43242E+12	1.20427E+13	1.78364E+13
714	1.74735E+12	5.10241E+12	8.40107E+12	9.08981E+12
715	5.09508E+12	5.54493E+12	5.8826E+12	1.2873E+13
716	5.31444E+12	3.29638E+12	7.01193E+12	1.59308E+13
717	2.48086E+12	1.89682E+12	8.72662E+12	1.34263E+13
718	7.90399E+11	4.35473E+12	7.07412E+12	1.09221E+13
719	2.02506E+12	4.58537E+12	5.06375E+12	9.79584E+12
720	1.06902E+12	3.57007E+12	7.45137E+12	9.92512E+12
721	2.06193E+12	1.88316E+12	8.03312E+12	1.20226E+13
722	4.83389E+12	1.85118E+12	6.86327E+12	1.32771E+13
723	5.41365E+12	5.76728E+12	8.77981E+12	1.04575E+13
724	4.77112E+12	2.93808E+12	7.73168E+12	1.04693E+13
725	2.62922E+12	3.01416E+12	5.92629E+12	1.06108E+13
726	3.35348E+12	3.97029E+12	5.04542E+12	8.81734E+12
727	6.56158E+12	4.38547E+12	5.02955E+12	6.52969E+12
728	5.48979E+12	5.04657E+12	5.1774E+12	6.7838E+12
729	2.25624E+12	4.0292E+12	4.97271E+12	3.99499E+12
730	1.20479E+12	3.16135E+12	7.13091E+12	6.43146E+12
731	2.56783E+12	2.78291E+12	6.89204E+12	9.52053E+12
732	4.2456E+12	3.19994E+12	3.95637E+12	8.55387E+12
733	1.95021E+12	3.0327E+12	5.62279E+12	5.84186E+12
734	1.28776E+12	1.17651E+12	7.16364E+12	5.35417E+12
735	5.83415E+11	1.78502E+12	6.56242E+12	5.63021E+12
736	5.72433E+11	3.38697E+12	5.68067E+12	5.55224E+12
737	1.3914E+12	3.6461E+12	5.70733E+12	4.97393E+12
738	1.26224E+12	3.03626E+12	4.49325E+12	4.7583E+12
739	2.50117E+11	2.99495E+12	3.74411E+12	5.99932E+12

740	2.52267E+12	2.27465E+12	4.90141E+12	6.01878E+12
741	4.48255E+12	1.33607E+12	7.16584E+12	5.2676E+12
742	3.37243E+12	6.82075E+11	7.46732E+12	5.21695E+12
743	3.43352E+12	6.38508E+11	4.50634E+12	5.35962E+12
744	1.26325E+12	2.22121E+12	2.95869E+12	5.90607E+12
745	3.89972E+12	3.4309E+12	1.92866E+12	4.81219E+12
746	7.45329E+12	3.69279E+12	2.95679E+12	3.10497E+12
747	4.45964E+12	2.75471E+12	6.14473E+12	2.35772E+12
748	1.15318E+12	6.22213E+11	5.19579E+12	2.6007E+12
749	2.28519E+12	1.3043E+12	2.14646E+12	4.25524E+12
750	2.34606E+12	3.36101E+12	1.49948E+12	2.96659E+12
751	2.08738E+12	4.03592E+12	2.94436E+12	2.39255E+12
752	5.24797E+12	2.47115E+12	3.4282E+12	8.56203E+12
753	2.76175E+12	3.14983E+12	2.2412E+12	5.94651E+12
754	1.2752E+12	2.81749E+12	1.99322E+12	4.29436E+12
755	2.62904E+12	2.43337E+12	1.79478E+12	4.43978E+12
756	5.11201E+12	2.07381E+12	2.02812E+12	4.00222E+12
757	2.89066E+12	7.45549E+11	4.29403E+12	1.33703E+12
758	1.46453E+12	1.7888E+12	2.44823E+12	4.677E+12
759	1.44725E+12	3.87137E+12	2.89617E+12	5.28145E+12
760	2.13511E+12	4.42836E+12	4.16207E+12	4.28605E+12
761	2.1907E+12	3.37244E+12	4.09926E+12	3.59466E+12
762	4.24092E+11	3.18652E+12	3.71594E+12	1.18686E+12
763	2.65989E+11	3.432E+12	1.89496E+12	1.46079E+12
764	8.30679E+11	2.61645E+12	1.09078E+12	1.5248E+12
765	2.94767E+12	1.33577E+12	1.39578E+12	1.17635E+12
766	5.51984E+12	7.9934E+11	1.9572E+12	1.17181E+12
767	5.18625E+12	2.96714E+12	3.40949E+12	4.23906E+12
768	1.80613E+12	2.50041E+12	2.5702E+12	5.22175E+12
769	4.53134E+11	1.42467E+12	2.49312E+12	3.3554E+12
770	1.40318E+12	7.15458E+11	3.1083E+12	1.3594E+12
771	1.98993E+12	3.53706E+11	3.694E+12	1.08293E+12
772	1.47931E+12	2.81047E+12	2.8519E+12	4.47956E+12
773	6.43132E+11	1.33081E+12	3.57645E+12	2.28882E+12
774	7.11512E+11	1.01026E+12	3.43863E+12	8.35699E+11
775	1.89458E+12	2.21959E+12	2.51498E+12	1.43191E+12
776	3.74141E+12	1.1432E+12	3.95796E+12	1.27291E+12
777	5.57048E+12	1.90409E+12	3.95045E+12	1.74606E+12
778	4.18461E+12	1.42698E+12	2.20735E+12	3.35054E+12
779	1.85889E+12	5.93431E+11	9.84386E+11	4.90702E+12
780	1.402E+12	9.06736E+11	1.54391E+12	3.9513E+12
781	4.5905E+12	4.31532E+12	3.18114E+12	8.15707E+11
782	8.6642E+12	3.15729E+12	3.82841E+12	3.80274E+12
783	5.09084E+12	1.72948E+12	3.80187E+12	4.88425E+12
784	3.89642E+11	1.06292E+12	2.24336E+12	4.47248E+12

785	2.90529E+11	1.3152E+12	1.99212E+11	4.96278E+12
786	2.92196E+11	2.04112E+12	14116607226	5.25211E+12
787	2.67023E+12	1.85719E+12	1.63027E+12	6.11886E+12
788	1.94186E+12	2.34963E+12	3.14123E+12	3.48213E+12
789	1.78356E+11	3.21653E+12	4.34546E+12	9.10456E+11
790	9.05909E+11	3.81477E+12	4.35938E+12	1.80678E+12
791	7.85404E+11	2.33395E+12	1.80126E+12	4.96587E+12
792	5.75509E+12	4.77713E+12	1.19796E+12	5.82364E+12
793	5.12082E+12	3.68087E+12	1.32893E+12	3.45742E+12
794	64329486842	3.70648E+11	1.93573E+12	5.61295E+11
795	3.06854E+11	1.28485E+12	4.37354E+12	206171472.2
796	2.42294E+12	1.84221E+12	3.06205E+12	3.87973E+12
797	3.48808E+12	1.35874E+12	2.27016E+12	8.98812E+12
798	3.83504E+12	2.10665E+12	1.70305E+12	7.04682E+12
799	3.15641E+12	4.61398E+12	2.01183E+12	9.18892E+11
800	8.75187E+11	4.48884E+12	5.46369E+12	2.75569E+12
801	4.94899E+12	1.04437E+12	2.68174E+12	5.31034E+12
802	6.60655E+12	1.06702E+12	7.00555E+11	3.50719E+12
803	4.469E+12	1.93628E+12	3.34577E+11	1.68924E+12
804	1.54413E+12	1.29126E+12	1.71251E+12	2.50307E+12
805	1.65185E+12	1.734E+12	5.59088E+12	3.07782E+11
806	1.48391E+12	2.95539E+12	2.17322E+12	1.61522E+12
807	4.67427E+12	1.79481E+12	6.96887E+11	1.62744E+12
808	5.84319E+12	1.25727E+12	1.03885E+12	8.51706E+11
809	1.12591E+12	4.2276E+12	4.24414E+11	1.92725E+12
810	5.26113E+12	9.97836E+11	3.6016E+12	3.13778E+12
811	2.84856E+12	9.81854E+11	2.13585E+12	2.1995E+12
812	4.49445E+12	1.63201E+12	2.08827E+12	1.36207E+12
813	1.00326E+13	1.87668E+12	3.57879E+12	1.44359E+12
814	7.77919E+12	3.71432E+12	1.56119E+12	1.28428E+12
815	5.0257E+12	4.60945E+12	4.30206E+12	1.26952E+12
816	2.90203E+12	3.9896E+12	3.16959E+12	1.74202E+12
817	1.40585E+12	3.9859E+12	1.92724E+12	1.55413E+12
818	2.1046E+12	4.40466E+12	3.13117E+12	7.36687E+11
819	9.95407E+12	1.74698E+12	5.04839E+12	1.70183E+12
820	4.19366E+12	1.16334E+12	2.5542E+12	5.31966E+12
821	1.14847E+12	4.39532E+12	3.67034E+12	3.66379E+12
822	2.10087E+12	6.02153E+12	4.28439E+12	9.42786E+11
823	2.16883E+12	2.8751E+12	0	1.31167E+12
824	1.05369E+12	5.64384E+12	0	3.99284E+11
825	4.78973E+11	2.39102E+12	2.32979E+12	1.37879E+12
826	5.02338E+11	1.78244E+12	3.90139E+12	1.88354E+12
827	3.11917E+12	3.77005E+12	3.59069E+12	1.11779E+12
828	8.6113E+12	2.56793E+12	1.13595E+12	73368961998
829	2.89534E+12	3.9496E+12	4.21542E+11	6.95704E+11

830	6.04387E+12	4.46727E+12	1.02979E+12	2.49037E+12
831	7.43326E+12	4.506E+12	2.25181E+12	2.53242E+12
832	2.01413E+12	3.78527E+12	4.276E+12	6.4214E+11
833	4.85434E+11	2.64047E+12	7.70532E+12	7.06519E+11
834	2.40018E+12	4.8156E+12	4.69564E+12	1.30167E+13
835	6.25971E+12	5.55479E+12	2.8718E+12	1.01307E+13
836	6.04144E+12	5.71057E+12	5.25038E+12	2.2818E+12
837	5.2941E+11	7.74919E+12	9.66613E+12	2.15501E+12
838	2.98368E+12	9.74438E+12	5.37324E+12	2.18207E+12
839	1.11277E+12	8.76732E+12	1.45607E+12	1.61476E+12
840	8.5026E+11	5.53549E+12	3.1961E+12	5.03682E+12
841	3.0098E+12	3.11416E+12	6.36858E+12	9.10082E+12
842	4.79609E+12	3.30435E+12	3.20023E+12	6.332E+12
843	8.90086E+12	4.89402E+12	3.96576E+12	4.30773E+12
844	4.51199E+12	3.46276E+12	2.96929E+12	3.59284E+12
845	1.33646E+12	2.4975E+12	1.35116E+12	4.77824E+12
846	4.01107E+12	3.06282E+12	1.58086E+12	7.5667E+12
847	3.03068E+12	1.35742E+12	7.26693E+12	1.46731E+13
848	3.51774E+12	3.56992E+11	3.66165E+12	1.33611E+13
849	6.4548E+12	3.18202E+12	1.18438E+12	1.10696E+13
850	6.89775E+12	4.55249E+12	4.05098E+11	1.12946E+13
851	2.2762E+12	1.78112E+12	7.00006E+11	1.39736E+13
852	2.06998E+12	4.75074E+12	3.88183E+12	1.1368E+13
853	3.28579E+12	2.79428E+12	7.0622E+12	1.82402E+13
854	8.33981E+12	4.90172E+12	8.74381E+12	1.88308E+13
855	1.04326E+13	7.26993E+12	7.65258E+12	1.54377E+13
856	3.00522E+12	1.39004E+12	3.93913E+12	1.85184E+13
857	2.57069E+12	6.14166E+12	5.58407E+12	1.75314E+13
858	8.28317E+12	6.6643E+12	3.35627E+12	1.64399E+13
859	8.27078E+12	4.7788E+12	3.62329E+12	1.67899E+13
860	2.06906E+12	4.33337E+12	5.59142E+12	1.74568E+13
861	5.73853E+12	6.54546E+12	8.16519E+11	1.59392E+13
862	4.02927E+12	8.94658E+12	1.69221E+11	2.21178E+13
863	6.02578E+12	8.01021E+12	3.07589E+12	2.05933E+13
864	1.20139E+13	4.51774E+12	6.33198E+12	1.62012E+13
865	1.36187E+13	2.22711E+12	7.09114E+12	1.56133E+13
866	2.1279E+12	7.0908E+12	6.40794E+12	1.35761E+13
867	9.69804E+12	2.95735E+12	3.60562E+12	1.21544E+13
868	9.3118E+12	3.00888E+12	5.90707E+12	1.56768E+13
869	4.71016E+12	6.57603E+12	9.44173E+12	1.94774E+13
870	1.64435E+13	5.52171E+12	5.79154E+12	1.24763E+13
871	6.77091E+12	8.68777E+12	6.5376E+12	8.7045E+12
872	1.98837E+12	1.19902E+13	5.48625E+12	8.3766E+12
873	1.98701E+12	1.08981E+13	5.7166E+12	8.56151E+12
874	4.77286E+12	5.92845E+12	9.07827E+12	7.91198E+12

875	1.26704E+13	1.09164E+12	1.2008E+13	1.42844E+13
876	5.86872E+12	1.47906E+12	5.82072E+12	1.01276E+13
877	2.23116E+12	3.57598E+12	1.77583E+12	1.11707E+13
878	3.90138E+12	5.65188E+12	4.02622E+12	1.63613E+13
879	5.93928E+12	6.05798E+12	1.2755E+13	1.63703E+13
880	9.3363E+12	3.36606E+12	4.58941E+12	9.48081E+12
881	1.36592E+13	3.04767E+12	1.43264E+12	5.15287E+12
882	1.70831E+13	1.99622E+12	3.29486E+12	3.29423E+12
883	1.46229E+13	2.59212E+12	9.43617E+12	5.80724E+12
884	1.05347E+13	1.10684E+13	1.32227E+13	1.45656E+13
885	1.39438E+13	5.57182E+12	7.42369E+12	1.38743E+13
886	9.67026E+12	2.38805E+12	7.32098E+12	9.07943E+12
887	4.96023E+12	4.74324E+12	9.86024E+12	6.04904E+12
888	7.42877E+12	1.00091E+13	1.00481E+13	5.70527E+12
889	4.48888E+12	8.16451E+12	4.6242E+12	1.16869E+13
890	1.82622E+12	3.03855E+12	8.66423E+12	5.608E+12
891	1.57145E+12	5.47563E+12	9.45523E+12	7.65385E+12
892	2.924E+12	1.21287E+13	9.19357E+12	1.67055E+13
893	5.85391E+12	1.29809E+13	1.11976E+13	1.62123E+13
894	1.8133E+13	7.55143E+12	1.08181E+13	3.4927E+12
895	2.00178E+13	5.79636E+12	6.71318E+12	2.69135E+12
896	1.84759E+13	6.22789E+12	3.67707E+12	8.57265E+12
897	1.72294E+13	5.29703E+12	4.76825E+12	1.62126E+13
898	1.24587E+13	5.00123E+12	1.16702E+13	1.53662E+13
899	1.27722E+13	1.35114E+13	1.16368E+13	5.47462E+12
900	1.78371E+13	1.10609E+13	1.32749E+13	4.90132E+12
901	1.69999E+13	6.70986E+12	1.65754E+13	6.35951E+12
902	6.5507E+12	1.14502E+13	1.31214E+13	3.41719E+12
903	6.19444E+12	9.72409E+12	4.161E+12	2.36287E+13
904	1.60229E+13	7.11061E+12	9.11713E+12	1.36207E+13
905	1.88182E+13	8.34727E+12	1.66883E+13	4.271E+12
906	1.17913E+13	1.00099E+13	1.56078E+13	3.37646E+12
907	5.85654E+12	3.2136E+12	3.96022E+12	8.38479E+11
908	1.93511E+12	7.9491E+12	3.61291E+12	1.30381E+12
909	3.21663E+12	5.57776E+12	1.20485E+13	5.06752E+12
910	6.95238E+12	7.01028E+12	2.03781E+13	6.85612E+12
911	7.7433E+12	1.52744E+13	1.69334E+13	4.62815E+12
912	3.87558E+12	3.02905E+12	2.51019E+12	1.38789E+13
913	2.20409E+12	4.34459E+11	7.66699E+12	7.62813E+12
914	1.00251E+13	3.91691E+12	1.22906E+13	8.96594E+12
915	2.15999E+13	8.8153E+12	1.32798E+13	1.81573E+13
916	1.87259E+13	1.09292E+13	1.52331E+13	1.66614E+13
917	8.32799E+12	6.27772E+12	7.59351E+12	3.74933E+12
918	3.62519E+12	3.03616E+12	9.07317E+12	1.55349E+12
919	4.39606E+12	9.69869E+12	1.39926E+13	3.20572E+12

920	5.59365E+12	1.96288E+13	1.61509E+13	5.60328E+12
921	1.68523E+12	3.23358E+12	1.78065E+13	1.68505E+13
922	1.17888E+12	0	6.00435E+12	8.14886E+12
923	2.49656E+12	1.35108E+12	6.24332E+12	1.1477E+13
924	3.22302E+12	2.37295E+12	1.18908E+13	1.99552E+13
925	1.29564E+12	1.23385E+12	4.30614E+12	1.68931E+13
926	5.30496E+12	9.06488E+12	1.53336E+12	5.74965E+13
927	5.2843E+12	1.28456E+13	2.50404E+12	5.58066E+13
928	4.41074E+12	9.89619E+12	6.99201E+12	4.60994E+13
929	5.40768E+12	4.53927E+12	9.49671E+12	4.89242E+13
930	4.33618E+12	9.22803E+12	4.29544E+12	4.73097E+13
931	2.27144E+12	5.28151E+12	1.5337E+12	4.75813E+13
932	1.02168E+13	1.12556E+13	5.59436E+12	4.63299E+13
933	1.62278E+13	1.33326E+13	1.29822E+13	4.40545E+13
934	6.37814E+12	32098868604	1.5356E+13	4.21875E+13
935	1.04167E+13	3.09502E+12	1.87237E+12	4.34519E+13
936	2.67239E+13	1.24647E+13	1.51658E+13	5.67281E+13
937	2.24651E+13	1.84373E+13	2.01959E+13	6.07693E+13
938	1.08865E+12	1.61009E+13	5.88767E+12	4.88458E+13
939	3.77326E+12	1.14231E+13	1.41238E+12	3.87228E+13
940	3.60029E+12	2.65639E+13	1.27603E+13	6.36023E+13
941	9.81827E+12	3.49769E+13	8.8572E+12	5.76375E+13
942	1.17939E+13	2.62289E+13	5.41538E+12	5.28133E+13
943	1.89556E+12	8.74297E+12	1.66296E+13	8.23907E+13
944	4.28226E+12	1.35134E+13	4.36631E+12	6.72785E+13
945	3.27154E+13	1.46375E+13	1.5089E+13	7.27067E+13
946	3.19874E+13	1.98524E+13	2.25326E+13	5.85426E+13
947	6.26933E+12	2.32837E+13	1.64976E+13	3.18347E+13
948	1.04293E+13	1.28078E+13	9.10465E+12	3.75543E+13
949	2.16399E+13	2.07181E+13	3.04137E+13	3.25282E+13
950	1.57219E+13	3.6525E+13	2.78832E+13	3.37331E+13
951	9.17389E+12	3.75469E+13	1.00919E+13	3.62357E+13
952	1.6334E+13	1.73303E+13	1.84217E+12	3.23567E+13
953	3.88352E+13	1.49013E+13	7.41438E+12	2.48447E+13
954	3.05153E+13	1.27928E+13	1.33565E+13	2.2839E+13
955	1.60469E+13	1.90892E+13	8.78807E+12	2.92191E+13
956	1.11316E+13	2.11292E+13	9.32827E+12	4.00102E+13
957	2.3165E+12	2.68514E+12	4.4381E+13	5.11384E+13
958	1.42804E+13	3.76795E+12	2.57526E+13	3.20435E+13
959	1.27838E+13	1.86842E+13	8.46132E+12	2.366E+13
960	7.4097E+12	2.29024E+13	2.98182E+12	3.72289E+13
961	1.19103E+13	5.76173E+12	5.69487E+12	6.29841E+13
962	1.5428E+13	1.90395E+12	2.32977E+12	2.81105E+13
963	2.07892E+13	3.63466E+12	1.17684E+12	3.68142E+13
964	5.1795E+13	5.10248E+12	5.76124E+12	5.84688E+13

965	7.76358E+13	1.00971E+13	2.08169E+13	5.09239E+13
966	3.60607E+13	2.25155E+13	5.78888E+13	8.46836E+12
967	9.17999E+12	7.53639E+12	1.50695E+13	2.06416E+13
968	3.73642E+12	3.74345E+12	2.15096E+13	2.80157E+13
969	1.2045E+13	9.64413E+12	3.76034E+13	2.47729E+13
970	1.97714E+13	1.63299E+13	1.73295E+13	1.87343E+13
971	1.60223E+13	2.3431E+13	1.7731E+13	2.09102E+13
972	4.16835E+13	3.28529E+13	2.66995E+13	2.58198E+13
973	3.67966E+13	3.65306E+13	2.2958E+13	2.10465E+13
974	5.53965E+12	2.61988E+13	1.20843E+13	1.06546E+13
975	7.75166E+12	1.59968E+13	2.01725E+13	1.13092E+13
976	1.27596E+13	2.29698E+13	1.32213E+13	1.45505E+13
977	1.01419E+13	2.21931E+13	2.07293E+13	1.81962E+13
978	8.85459E+12	1.26491E+13	3.57497E+13	2.1609E+13
979	1.38688E+13	3.00957E+12	3.5987E+13	2.34936E+13
980	9.98355E+12	2.19078E+13	1.31002E+13	2.03531E+13
981	4.53478E+12	1.55561E+13	1.53341E+13	1.94471E+13
982	4.79866E+12	2.87582E+13	2.1244E+13	1.24093E+13
983	9.55733E+12	4.8961E+13	2.23249E+13	1.46089E+13
984	9.47462E+12	2.04736E+13	2.83583E+13	5.92229E+13
985	7.39095E+12	3.64047E+13	6.43534E+12	2.39548E+13
986	2.43387E+13	4.22246E+13	2.9188E+13	2.85571E+13
987	3.24875E+13	3.55838E+13	4.66655E+13	4.30888E+13
988	9.57849E+12	2.91493E+13	1.46018E+13	1.78343E+13
989	7.82167E+12	2.87046E+13	1.27255E+13	1.73583E+13
990	5.0514E+13	4.3516E+13	3.78322E+13	3.95884E+13
991	6.27887E+13	3.56918E+13	3.85175E+13	3.83879E+13
992	3.5028E+13	1.47939E+13	1.47811E+13	2.2439E+13
993	2.55896E+13	3.06248E+13	2.46199E+13	5.30773E+13
994	3.98355E+13	3.66963E+13	7.92303E+13	2.16625E+13
995	4.00214E+13	1.95702E+13	8.00937E+13	3.69937E+13
996	2.59718E+13	9.19879E+12	3.44641E+13	5.53451E+13
997	4.00223E+12	2.57053E+13	3.38127E+12	1.25707E+13
998	2.67236E+13	1.17809E+13	2.44228E+13	9.0738E+12
999	6.00301E+13	1.19145E+13	3.76734E+13	3.99156E+13
1000	6.2006E+13	2.80539E+13	5.23092E+13	4.65362E+13
