# Supplementary material for: Attractiveness of Green Stink Bugs Nezara spp. to Ultraviolet-Based Multichromatic Light Traps: Synergistic Effects of Ultraviolet and Blue Light
Source: Insects. 2026 Mar 3;17(3):270. doi: 10.3390/insects17030270 (PMC13026388; doi:10.3390/insects17030270)
Supplement: Supplementary file 1 [file insects-17-00270-s001.zip › Table S2.pdf]

Supplementary Table S2. Raw captured data of insects attracted to UV-based multichromatic lights.

Nezara viridula

Year	Date	Location	Light source				
			UV+Blue	UV+Green	UV+Orange	UV+Red	UV84
2024	8/26-8/30	Yamaguchi	2	4	1	3	1
2024	8/31-9/2	Yamaguchi	0	0	0	1	1
2024	9/3-9/6	Yamaguchi	1	2	1	0	0
2024	9/7-9/9	Yamaguchi	1	1	1	0	1
2024	9/10-9/13	Yamaguchi	1	3	0	1	3
2024	9/14-9/17	Yamaguchi	9	6	0	0	2
2024	9/18-9/20	Yamaguchi	2	2	0	1	0
2024	9/21-9/24	Yamaguchi	1	5	0	0	0
2024	9/25-9/27	Yamaguchi	2	4	2	1	0
2024	9/28-10/1	Yamaguchi	12	0	6	2	2
2024	10/2-10/4	Yamaguchi	0	0	0	0	0
2024	10/5-10/8	Yamaguchi	3	0	2	3	3
2024	10/9-10/12	Yamaguchi	6	3	1	0	1
2024	10/13-10/16	Yamaguchi	3	3	6	1	1

Nezara antennata

Year	Date	Location	Light source				
			UV+Blue	UV+Green	UV+Orange	UV+Red	UV84
2019	7/30-8/2	Niigata	13	7	0	3	5
2019	8/3-8/5	Niigata	7	25	4	2	7
2019	8/6-8/8	Niigata	6	6	4	3	4
2019	8/9-8/11	Niigata	2	1	1	1	0
2019	8/12-8/14	Niigata	1	2	0	0	2
2019	8/15-8/17	Niigata	2	1	0	1	0
2019	8/18-8/20	Niigata	3	0	0	1	0
2019	8/21-8/23	Niigata	1	0	0	1	0
2019	8/24-8/26	Niigata	1	0	0	0	0
2019	8/27-8/29	Niigata	0	0	0	0	0
2019	8/30-9/1	Niigata	0	0	1	0	0
2019	9/2-9/4	Niigata	0	1	0	0	0
2019	9/5-9/7	Niigata	0	1	0	0	1

Piezodorus hybneri

Year	Date	Location	Light source				
			UV+Blue	UV+Green	UV+Orange	UV+Red	UV84
2024	8/26-8/30	Yamaguchi	0	0	0	0	0
2024	8/31-9/2	Yamaguchi	1	2	0	1	1
2024	9/3-9/6	Yamaguchi	1	1	3	3	1
2024	9/7-9/9	Yamaguchi	1	1	2	0	0
2024	9/10-9/13	Yamaguchi	0	6	2	1	1

2024	9/14-9/17	Yamaguchi	11	4	0	0	0
2024	9/18-9/20	Yamaguchi	3	0	1	0	2
2024	9/21-9/24	Yamaguchi	0	0	0	0	0
2024	9/25-9/27	Yamaguchi	0	0	0	0	0
2024	9/28-10/1	Yamaguchi	0	0	0	0	0
2024	10/2-10/4	Yamaguchi	0	0	0	0	0
2024	10/5-10/8	Yamaguchi	0	1	0	0	0
2024	10/9-10/12	Yamaguchi	1	2	0	1	0
2024	10/13-10/16	Yamaguchi	0	0	0	0	0

Pleuroptya ruralis

Year	Date	Location	Light source				
			UV+Blue	UV+Green	UV+Orange	UV+Red	UV84
2019	7/30-8/2	Niigata	1	1	0	0	2
2019	8/3-8/5	Niigata	0	0	0	0	0
2019	8/6-8/8	Niigata	0	0	0	0	1
2019	8/9-8/11	Niigata	0	0	0	0	0
2019	8/12-8/14	Niigata	0	0	0	0	0
2019	8/15-8/17	Niigata	3	2	0	0	0
2019	8/18-8/20	Niigata	0	4	0	2	0
2019	8/21-8/23	Niigata	4	3	0	3	1
2019	8/24-8/26	Niigata	8	10	5	3	2
2019	8/27-8/29	Niigata	2	3	4	2	1
2019	8/30-9/1	Niigata	1	0	1	0	1
2019	9/2-9/4	Niigata	0	2	2	0	0
2019	9/5-9/7	Niigata	1	3	0	0	0

Anomala albopilosa

Year	Date	Location	Light source				
			UV+Blue	UV+Green	UV+Orange	UV+Red	UV84
2024	8/26-8/30	Yamaguchi	59	10	27	39	5
2024	8/31-9/2	Yamaguchi	28	4	4	2	4
2024	9/3-9/6	Yamaguchi	20	8	11	0	2
2024	9/7-9/9	Yamaguchi	3	0	11	1	1
2024	9/10-9/13	Yamaguchi	3	3	8	3	6
2024	9/14-9/17	Yamaguchi	1	0	0	2	1
2024	9/18-9/20	Yamaguchi	0	0	1	0	0
2024	9/21-9/24	Yamaguchi	0	0	0	0	1
2024	9/25-9/27	Yamaguchi	0	0	0	0	1
2024	9/28-10/1	Yamaguchi	0	0	0	0	0
2024	10/2-10/4	Yamaguchi	0	0	0	0	0
2024	10/5-10/8	Yamaguchi	0	0	0	0	0
2024	10/9-10/12	Yamaguchi	0	0	0	0	0
2024	10/13-10/16	Yamaguchi	0	0	0	0	0

Anomala rufocuprea

Year	Date	Location	Light source				
			UV+Blue	UV+Green	UV+Orange	UV+Red	UV84
2019	7/30-8/2	Niigata	14	10	4	8	17
2019	8/3-8/5	Niigata	7	2	4	7	5
2019	8/6-8/8	Niigata	1	2	4	1	4
2019	8/9-8/11	Niigata	5	3	3	1	4
2019	8/12-8/14	Niigata	6	0	1	0	1
2019	8/15-8/17	Niigata	6	2	2	1	0
2019	8/18-8/20	Niigata	5	0	0	2	2
2019	8/21-8/23	Niigata	3	1	2	0	1
2019	8/24-8/26	Niigata	1	0	1	1	2
2019	8/27-8/29	Niigata	3	2	1	1	0
2019	8/30-9/1	Niigata	0	2	1	0	0
2019	9/2-9/4	Niigata	0	0	3	1	0
2019	9/5-9/7	Niigata	2	4	1	1	1
