# Supplementary material for: Attractiveness of Green Stink Bugs Nezara spp. to Ultraviolet-Based Multichromatic Light Traps: Synergistic Effects of Ultraviolet and Blue Light
Source: Insects. 2026 Mar 3;17(3):270. doi: 10.3390/insects17030270 (PMC13026388; doi:10.3390/insects17030270)
Supplement: Supplementary file 1 [file insects-17-00270-s001.zip › Table S3.pdf]

Supplementary Table S3. Raw captured data of insects attracted to monochromatic UV, blue lights and combined UV and green light.

Nezara viridula

Year	Date	Location	Light source		
			2Blue	UV+Blue	2UV
2023	7/25-8/1	Yamaguchi	0	0	0
2023	8/2-8/8	Yamaguchi	0	0	0
2023	8/9-8/15	Yamaguchi	0	0	0
2023	8/16-8/22	Yamaguchi	0	2	0
2023	8/23-8/29	Yamaguchi	2	4	5
2023	8/30-9/5	Yamaguchi	0	6	6
2023	9/6-9/12	Yamaguchi	0	2	1
2023	9/13-9/19	Yamaguchi	1	16	2
2023	9/20-9/26	Yamaguchi	5	26	7
2023	9/27-10/3	Yamaguchi	0	13	5

Nezara antennata

Year	Date	Location	Light source		
			2Blue	UV+Blue	2UV
2022	7/20-7/22	Niigata	0	0	2
2022	7/23-7/25	Niigata	0	1	1
2022	7/26-7/27	Niigata	0	4	1
2022	7/28-7/29	Niigata	0	9	4
2022	7/30-8/1	Niigata	5	11	2
2022	8/2-8/3	Niigata	0	4	7
2022	8/4-8/5	Niigata	0	1	0
2022	8/6-8/8	Niigata	1	6	0
2022	8/9-8/10	Niigata	0	6	1
2022	8/11-8/12	Niigata	1	2	0
2022	8/13-8/15	Niigata	0	0	0
2022	8/16-8/17	Niigata	1	8	0
2022	8/18-8/19	Niigata	0	1	0
2022	8/20-8/22	Niigata	0	1	0
2022	8/23-8/24	Niigata	1	2	0
2022	8/25-8/26	Niigata	0	0	0
2022	8/27-8/29	Niigata	0	1	0
